# Ethical and Practical Considerations of Physicians and Nurses on Integrating Artificial Intelligence in Clinical Practices in Saudi Arabia: A Cross-Sectional Study

**DOI:** 10.3390/nursrep15090309

**Published:** 2025-08-25

**Authors:** Abdulaziz Rashed Alsaedi, Maisam Elfaki Haddad, Roaa Matouq Khinkar, Sumayyah Mohammed Alsharif, Anhar Abdelwahab Elbashir, Ahlam Ali Alghamdi

**Affiliations:** 1 Medical Services, Prince Mohammed Bin Abdulaziz Hospital, National Guard Health Affairs, Madinah 40740, Saudi Arabia; 2 College of Management, University of Midocean, Matelec Street, P.O. Box 684, Moroni 98123, Comoros; 3 Primary Healthcare Center, Prince Mohammed Bin Abdulaziz Hospital, National Guard Health Affairs, Madinah 40740, Saudi Arabia; haddadma@mngha.med.sa (M.E.H.); shariefsu@mngha.med.sa (S.M.A.); 4 Department of Pharmacy Practice, Faculty of Pharmacy, King Abdulaziz University, Jeddah 21589, Saudi Arabia; rkhinkar@kau.edu.sa; 5 Sudanese Consortium for Surgical Development Fellowship Program, Khartoum 11115, Sudan; anharelbashir@gmail.com; 6 Dental Department, King Fahad Hospital, Jeddah 23325, Saudi Arabia; aalghamdi896@moh.gov.sa

**Keywords:** artificial intelligence, clinical practices, ethics, practical considerations, healthcare professionals

## Abstract

**Background/Objectives**: The emergence of artificial intelligence (AI) has revolutionized the healthcare industry. However, its integration into clinical practices raises ethical and practical concerns. This study aims to explore ethical and practical considerations perceived by physicians and nurses in Saudi Arabia. **Methods**: It employed a cross-sectional design with 400 physicians and nurses, using a pre-established online questionnaire. Descriptive data were analyzed through means and standard deviations, while inferential statistics were performed using the independent samples *t*-test. **Results**: Most participants were male (57%) and physicians (73.8%), with most employed in governmental organizations (87%). The participants’ use and awareness of AI was low, as 34.0% said they had never used it, but 74.5% of respondents were willing to use AI in clinical practices. Also, 80.5% of participants were aware of the AI benefits, and 71.0% had background knowledge about the ethical concerns related to AI’s implementation in their clinical practices. Moreover, (62.0%) of respondents recognized the applicability of AI in their specialty. Key findings revealed significant concerns: participants perceived a lack of skills to effectively utilize AI in clinical practice (mean = 4.04) and security risks such as AI manipulation or hacking (mean = 3.83). The most pressing ethical challenges included AI’s potential incompatibility with all populations and cultural norms (mean = 3.90) and uncertainty regarding responsibility for AI-related errors (mean = 3.84). **Conclusions**: These findings highlight substantial barriers that hinder the effective integration of AI in clinical practices in Saudi Arabia. Addressing these challenges requires leadership support, specific training initiatives, and developing practical strategies tailored to the local context. Future research should include other healthcare professionals and qualitatively explore further underlying factors influencing AI adoption.

## 1. Introduction

Artificial intelligence (AI) has recently been at the forefront of global technological innovation, obtaining headlines all over the world [1]. It has evolved as a result of the growing use of automation and technology to simulate human abilities in critical thinking and decision-making processes, leading to the emergence of AI [2]. AI was first developed in 1950, when Alan Turing raised his widely discussed question, “Can machines think?” [3].

Historically, the rules-based systems were found as the inception of a machine intelligence that does whatever is set only by programmers, and then they were advanced over time to more sophisticated algorithms that have the ability and capability to imitate the human brain in performing countless and complex tasks, referred to today as AI [4]. Practically, AI consists of diverse techniques, including machine learning (ML), deep learning (DL), natural language processing (NLP), and computer vision (CV) [4,5]. The features offered by these applications include, but are not limited to, visual perceptions, speech recognition, decision-making, and translation between languages [6].

Since its evolution, AI has been subsequently adopted and employed by many stakeholders, including manufacturing, e-commerce, banking, education, and healthcare [6]. In healthcare, for example, the adoption of AI has been attributed to improving patient outcomes, including the diagnosis of medical conditions, development of customized treatment plans, provision of preventive interventions, and invention of drugs [7]. As a result, a notable transformation has been experienced in the provision of medical services such as medicine, radiology, pathology, and dermatology [8]. Apart from this, it has gained a crucial role in supporting medical practices in the prognosis of conditions, as well as the invention of novel therapies.

AI applications are being applied more and more to improve clinical and administrative performance, which then in turn improves overall patient outcomes. To cope with stress related to textual data entered in medical records, for example, NLP is being utilized [9]. Additionally, CV technology has improved the process of reading radiological images by influencing their interpretation and analysis, while ML has assisted with data analysis and providing health professionals’ insights [10].

Even though adopting AI has transformed the healthcare industry and produced exceptional performance in terms of provided services, a review of the literature revealed several challenges that hinder its full integration and application in clinical practice. The study of Aldhafeeri [11] was conducted in the Saudi Arabian context. It reported that the highest-scored concerns were uncertainty about the outcome of AI systems, patients, and reliance on AI. Meanwhile, the study of Elnaggar et al. [12] reported that replacing the healthcare providers’ jobs and patient privacy issues were more important concerns perceived by the study respondents.

Despite the substantial amount of literature targeting the ethical challenges associated with AI integration into clinical practice, there is a dearth of solid guidelines and standardized frameworks or international collaboration that fully tackles healthcare AI-related ethical and practical issues. Therefore, further assessment of such challenges at the global level is warranted to promote the appropriate application of AI in healthcare. Additionally, proactive assessment of AI integration-related ethical concerns will assist in closing the current knowledge gap and guaranteeing the provision of safe, reliable, and high-quality patient care [13,14]. To ameliorate this matter, this study aimed to identify the ethical and practical considerations from physicians’ and nurses’ perspectives regarding AI integration in clinical practices in Saudi Arabia.

## 2. Materials and Methods

### 2.1. Study Design

A cross-sectional study was carried out including Saudi Arabian practicing physicians and nurses, with the exclusion of dentists and those assigned administrative responsibilities. Sample size was calculated using the formula n = Z^2^P (1 − P)/d^2^, where Z = 1.96 for a 95% confidence level, *p* = 0.5 for an assumed proportion, and d = 0.05 as the margin of error. Accordingly, the estimated sample size was 385 respondents.

### 2.2. Questionnaire Development and Validation

A close-ended online questionnaire was developed by the research team members and was voluntarily validated by three independent subject-matter experts to ensure its validity. It included 26 questions divided into four sections: demographics (4 items), practitioners’ experience with AI (5 items), practitioners’ concerns about the integration of AI in clinical practice (10 items), and ethical challenges of integrating AI into clinical practice (7 items) (Supplementary Materials).

Post-receipt of the IRB approval, the questionnaire was randomly piloted on 30 selected practitioners from different healthcare institutions who met the inclusion criteria. Based on their feedback, comments, and suggestions, the research tool was modified. This step aimed to ensure the face validity of the developed questionnaire and to measure the internal consistency (reliability) using Cronbach’s alpha coefficient. Accordingly, the internal consistency was found to be good, with an overall Cronbach’s alpha score of 0.866.

### 2.3. Conceptual Framework

While the current study employed a descriptive design, aiming to present a population’s perspectives on the integration of AI in clinical practices without testing theoretical models or any form of relationships, a conceptual framework is omitted. Therefore, the conceptual framework was not fundamental for achieving the study’s objectives.

### 2.4. Data Collection

Following the obtaining of IRB approval, the data collection process commenced. Questionnaires were distributed online via Google Forms on social media, including X (formerly Twitter), WhatsApp, Telegram, and LinkedIn platforms. All collected responses were de-identified through anonymous participation, stored, and kept confidential. Participation was voluntary, where the respondent had to agree before answering questions. Nevertheless, participants had the right to withdraw at any point, and their responses would then be excluded immediately.

### 2.5. Statistical Analysis

Questionnaires were analyzed using the Statistical Package for Social Sciences (SPSS) version 29. Descriptive data were analyzed using the central tendency, including frequency, mean, and standard deviation (SD), while the inferential data were analyzed using the independent samples *t*-test.

## 3. Results

### 3.1. Descriptive Statistics

Out of the distributed questionnaires, 400 participants responded to the questionnaires, representing diverse sites of healthcare institutions in Saudi Arabia. It was difficult to calculate the response rate where there was no identified population frame. Of these responses, n = 228 (57.0%) were male, and the remaining were female, with n = 172 (43.0%). In terms of the position, n = 295 (73.8%) were physicians, while nurses represented a lower percentage, with n = 105 (26.3%). Most of the respondents work in governmental hospitals, n = 348 (87.0%), while the remaining work at private and military hospitals, n = 30 (7.5%) and n = 22 (5.5%). Finally, the results showed that the majority of participants were from urban areas, with n = 352 (88.0%), and the others were from rural areas with, n = 48 (12.0%) (Table 1).

As for the participants’ use and awareness of AI, the study revealed that the overall frequency was low; n = 136 (34.0%) had never used it. The majority of respondents were willing to use AI in clinical practices, with n = 298 (74.5%). In terms of potential benefits and concerns, n = 322 (80.5%) of participants were aware of AI’s benefits, while n = 284 (71.0%) had background knowledge about AI’s ethical concerns in relation to its implementation in their clinical practices. Moreover, most respondents recognized the applicability of AI in their specialty, with n = 248 (62.0%) (Table 1).

As for the concerns related to AI integration in clinical practice, the highest mean scores were associated with a lack of adequate skills for effective utilization of AI and manipulation of AI-based systems by a third party, with mean scores of 4.04 and 3.83, respectively. On the other hand, the lowest mean scores were related to diminished roles of physicians and nurses, and increased waste related to overutilization of healthcare services, 3.24 and 3.22, respectively (Table 2).

As part of the ethical challenges related to AI integration in clinical practice, the highest mean scores were first associated with the inapplicability of specific AI algorithms to diverse populations and cultural norms and then the accountability and responsibility issues related to potential decision errors resulting from the utilization of AI, 3.90 and 3.84, respectively. The lowest mean scores were linked to the acquisition of informed consent from patients in regard to AI functionalities and potential delivery of inequitable patient care as a result of AI biases, 3.55 and 3.28, respectively (Table 3).

### 3.2. Independent t-Test: Difference in the Mean Score of AI Concerns by Position

As reflected in Table 4 and Table 5, an independent samples *t*-test was conducted to measure the difference between physicians and nurses regarding the integration of AI in clinical practices. The results showed that there was no significant difference in the mean scores between physicians (M = 3.52, SD = 0.57) and nurses (M = 3.56, SD = 0.57); *t* (398) = −0.64, *p* = 0.522, 95% CI [−0.17, 0.09]. Levene’s test indicated that the assumption of equal variances was met: F = 0.17, and *p* = 0.683 (Table 4 and Table 5). Meanwhile, Cohen’s d indicates a negligible effect size, with a score of (−0.073). The 95% confidence interval for Cohen’s d was [−0.296, 0.150], suggesting that the true effect falls within this range (Table 6).

### 3.3. Independent t-Test: Difference in the Mean Score of AI Ethical Challenges by Position

The independent samples *t*-test showed that there was no significant difference in the mean scores for physicians (M = 3.66, SD = 0.65, n = 295) and nurses (M = 3.69, SD = 0.65, n = 105); *t* (398) = −0.39, *p* = 0.697, 95% CI [−0.18, 0.12]. Levene’s test for equality of variances was not significant (F = 0.18, *p* = 0.668), indicating that the assumption of equal variances was met (Table 7 and Table 8). While the Cohen’s d indicates a negligible effect size with a score of (−0.044). The 95% confidence interval for Cohen’s d was [−0.267, 0.179], suggesting that the true effect falls within this range (Table 9).

## 4. Discussion

AI has a promising impact on the provision of healthcare services. However, to ensure its effective and reliable operability in healthcare, AI must be comprehensively assessed and evaluated. This cross-sectional study has addressed physicians’ and nurses’ perspectives regarding the ethical and practical considerations of AI integration in clinical practices. Their feedback represents healthcare providers’ perspectives on the use of AI in streamlining processes and supporting medical decision-making. Overall, the study reveals that both physicians and nurses lack the skills to use AI effectively, and it emphasizes the fact that AI algorithms developed for specific populations might not be suitable for another.

Participants in the study reported conflicting findings. Most of them do not apply AI in clinical practices, even though they are willing to do so. This could be an indication of their enthusiasm and level of knowledge. However, it appears that the organizations they practiced in did not encourage or support healthcare providers in using this technology to provide medical care. As a result, the discrepancy between willingness and actual practice is explicit. Thus, the excitement of physicians and nurses might promote the use of AI in clinical fields.

When it comes to the AI-related concerns from the perspective of healthcare professionals, the result of our study reveals that physicians and nurses believe that the lack of essential skills was attributed to the ineffective adoption of AI in clinical fields. Lack of skills might lead to the loss of the opportunity of leveraging AI in providing better and safer clinical care. This implies that there is a demand for fostering an organizational culture that supports the integration of AI in healthcare through equipping practitioners with the required knowledge and training to improve AI literacy and implementation [15]. This might be because AI is still a relatively new technology in the medical field and is not being used extensively in hospitals. This result aligns with Naik et al.’s study [16], which emphasized the importance of clinicians’ competencies and proficiency in augmenting the benefits gained from AI. Thus, for successful adoption of AI-supported clinical care, healthcare leadership should take a multidimensional approach where AI governance is ensured through cultivating a culture of AI literacy and integration, focusing on strategic planning, providing mentoring and resources, and investing in training and development. In addition, to meet the potential demand of the healthcare sectors, leaders in healthcare education should proactively equip the future healthcare workforce with essential skills to fully harness AI potential. This is consistent with the study of Hamd et al. [17], which recommends teaching medical students about the use of AI as part of the medical school curriculum.

Another key finding for the study is the potential manipulation of the AI database by a third party. Most participants expressed their worries about the quality of data released from AI-based systems that, as a result, could lead to poor clinical outcomes and, eventually, patient safety issues. From the technical point of view, these AI algorithms are trained using massive databases [18]. Consequently, inadequate data may be incorporated and have an impact on the provision of healthcare services, which could result in substandard clinical outcomes. According to the study of Goktas and Grzybowski [19], data themselves have the potential to negatively influence the clinical recommendations, leading to suboptimal diagnosis of medical conditions, therapeutic plans, and overall patient health outcomes. Likewise, biased or manipulated data were the most common concerns perceived by healthcare professionals [18]. Moreover, the studies of Obermeyer et al. [20] and Hantel et al. [21] support our findings. It reported that AI algorithms had racial bias affecting the prediction of health status among White and Black patients. Inadvertently, AI algorithms may have potential biases that could lead to inequitable diagnosis or treatment [22]. In dermatology, as an example, AI may exhibit bias based on race when determining skin disorders among people with darker skin colors in comparison to those with lighter-colored types [23]. In rare cases of dermatology, as discussed by Refolo et al. [24], given the lack of adequate image criteria along with the absence of standardized guidelines, the data utilized for AI training can lead to biased AI algorithms and consequently expose patients to system vulnerabilities, including inequitable care.

Additionally, the study showed that physicians and nurses do not believe that AI can accurately understand patients’ medical conditions. Accordingly, they believe that management and diagnosis of medical conditions by physicians are more reliable than such technology. In contrast, the study of Elendu et al. [22] highlighted that the introduction of AI in healthcare has refined the role of practitioners in one way or another. Apart from this, AI may facilitate and streamline the process of analyzing huge volumes of data and identifying patterns that challenge physicians and other healthcare providers [25]. Therefore, failure to appropriately understand patients’ medical status might expose them to diagnostic errors and potential harm [24]. In addition to that, the study of Gundlack et al. [26] reported the inability of AI to simulate the essential characteristics that humans have, such as patient–provider rapport in general, and empathy, as well as emotional intelligence, in particular, can dramatically impact health outcomes. Despite the rapidly increasing introduction of AI in clinical practices, the study of Pressman et al. [27] demonstrated the inability of AI to substitute physicians’ knowledge and judgment. This supports the fact that AI-based tools and systems must be leveraged to help with and improve the provision of healthcare services but not to replace clinicians [28].

Regarding the challenges of AI, the current study reported three major issues as perceived by participants. It showed that algorithms programmed in one culture may not be appropriate for the other one. This is like Tilala et al.’s study findings [25], which concluded that a customized AI for one group might not be suitable for another, particularly if two populations have different cultures and norms. Due to such differences, AI might provide inaccurate outcomes. For example, AI algorithms that are widely disseminated and utilized in the US to determine required clinical care for African American patients give different information for Caucasian patients, even if they have the same score [29]. This is supported by the findings of Monteith et al.’s study [30], which reported that AI models do not perform well when they are deployed in settings where the characteristics of their population differ from the characteristics used for the training.

Another key point to highlight is the need to protect patient privacy and ensure data security in healthcare. According to the participants in this study, the privacy and security issues that are associated with the integration of AI-based systems are still not adequately addressed. This matter has been raised by many studies, such as Weiner et al. [31], He et al. [32], and Currie and Hawk [33]. It is evident that the availability of patients’ data is crucial in training and testing AI models. Therefore, the lack of such data leads to limited training and eventually hinders the potential benefits of AI tools [6]. The tendency of AI systems to gather and analyze enormous volumes of patient data makes them a desirable target for hackers, with possible consequences including identity theft, monetary loss, harm to an individual’s reputation, and loss of trust [8]. This emphasizes the need for strong data protection protocols.

According to participants’ perspectives, this study reports that the cross-border issue is one of the main challenges. It is believed that information exchange and international collaboration might necessitate the adoption of different regulations and standards of care. The study of Lewin et al. [34] raised the concerns of sharing patient information where this cutting-edge technology should not affect or breach individuals’ privacy. However, previous studies emphasized that sharing patient information is fundamental to feeding AI models with real data. These models have the potential to assist healthcare providers in advancing precision medicine and optimizing care plans [5]. Despite the urgency for regulatory rules to govern the disclosure of patient data at the national and international levels, the systematic review conducted by Karimian et al. [35] reported a lack of a comprehensive ethical framework for AI in healthcare. Nevertheless, sharing patient medical information mitigates potential biases and ensures open access to these data by developers to enhance the process of testing and training AI models [36].

Our study had several strengths, including a sample from different healthcare settings and locations in Saudi Arabia, thus increasing the generalizability of the study findings. In alignment with AI integration as part of the Saudi Healthcare Sector Transformation Program (HSTP), this study is well-timed and is considered the first one at the national level in terms of exploring the ethical and practical issues related to AI integration. Apart from this, notable physicians’ responses signal the urgency for policymakers to accelerate AI integration as part of the Saudi HSTP. Another strength was the thorough analytical statistics used. This study conducted independent samples *t*-tests to figure out the differences between physicians’ and nurses’ perspectives regarding the AI concerns and ethical challenges of its integration into clinical practices.

The study has four limitations. Firstly, the current study targeted only physicians and nurses. Involving dentists and allied health professionals might enrich the findings with insightful information. Secondly, the number of received responses from participants is somewhat small compared to the number of physicians and nurses in the Kingdom of Saudi Arabia. Thirdly, the study was focused on quantitative data only, using predetermined concerns and challenges. If it involved individual semi-structured interviews, it might have explored more sensitive factors related to the effective integration of AI in clinical practices. Fourthly, years of experience were not studied in the current study, which may affect the awareness and willingness of healthcare providers to integrate AI into clinical practices.

## 5. Conclusions

This study focuses on identifying the physicians’ and nurses’ concerns about the integration of AI in clinical practices and the associated ethical considerations in Saudi Arabia. The findings indicate that participants raised some concerns that avert the effective adoption of AI, particularly in the clinical field. Furthermore, these results contribute to a growing body of evidence supporting the governments and healthcare decision-makers in support of the integration of AI in healthcare. While this study was limited to physicians and nurses, its insights suggest that similar studies could benefit dentists and other allied health specialists. Therefore, leadership support is a cornerstone for building a culture of AI integration where AI is incorporated as part of the workflows and daily roles. This can be achieved through leveraging AI literacy via weaving AI into the strategic and operational plans and providing education and training, as well as securing resources for reliable AI implementation and integration. Furthermore, qualitative research should be conducted to explore other challenges and practical recommendations. Where physicians’ and nurses’ years of experience were not considered, future studies could explore whether experience level affects attitudes toward AI adoption. Ultimately, mitigating these challenges is essential to maximize the benefits from AI and enhance both patient safety and patient experience.

## Figures and Tables

**Table 1:** Demographic statistics of study participants.

	N (%)
**Gender**	
Male	228 (57.0%)
Female	172 (43.0%)
**Position**	
Physician	295 (73.8%)
Nurse	105 (26.3%)
**Type of institution**	
Governmental hospitals	348 (87.0%)
Military clinic	22 (5.5%)
Private practice	30 (7.5%)
**Location**	
Urban area	352 (88.0%)
Rural area	48 (12.0%)
**Frequency of artificial intelligence use in clinical practice**	
Daily	86 (21.5%)
Weekly	50 (12.5%)
Occasionally	128 (32.0%)
Never	136 (34.0%)
**Are you willing to use “artificial intelligence” tools in your clinical practice?**	
Yes	298 (74.5%)
No	102 (25.5%)
**Are you aware of the potential benefits of using artificial intelligence?**	
Yes	322 (80.5%)
No	78 (19.5%)
**Are you aware of the potential concerns of using artificial intelligence?**	
Yes	284 (71.0%)
No	116 (29.0%)
**Do you know there is an area for use of AI in your specialty?**	
Yes	248 (62.0%)
No	152 (38.0%)

**Table 2:** Physicians’/nurses’ concerns about the integration of artificial intelligence in clinical practice.

	Strongly Disagree	Disagree	Neutral	Agree	Strongly Agree	Mean	Rank
1. Artificial intelligence might not understand complex medical conditions as accurately as physicians and nurses do.	6 (1.5%)	42 (10.5%)	90 (22.5%)	170 (42.5%)	92 (23.0%)	3.75	3
2. Artificial intelligence could reduce the roles that physicians/nurses traditionally play.	26 (6.5%)	86 (21.5%)	90 (22.5%)	162 (40.5%)	36 (9.0%)	3.24	9
3. Physicians/nurses might feel more stressed because of the additional demands of using technology/AI.	16 (4.0%)	100 (25.0%)	98 (24.5%)	136 (34.0%)	50 (12.5%)	3.26	8
4. Artificial intelligence could potentially weaken the relationship between patients and their treating team.	20 (5.0%)	92 (23.0%)	68 (17.0%)	154 (38.5%)	66 (16.5%)	3.39	7
5. Not all physicians/nurses have the adequate skills to use artificial intelligence effectively.	2 (0.5%)	14 (3.5%)	62 (15.5%)	212 (53.5%)	110 (27.5%)	4.04	1
6. There is a concern that artificial intelligence-based systems could be manipulated from outside (third party, hackers, etc.)	6 (1.5%)	22 (5.5%)	106 (26.5%)	166 (41.5%)	100 (25.0%)	3.83	2
7. Artificial intelligence will worsen problems in healthcare such as overutilization of laboratory testing, overdiagnosis, and overtreatment.	16 (4.0%)	98 (24.5%)	118 (29.5%)	120 (30.0%)	48 (12.0%)	3.22	10
8. The use of artificial intelligence may negatively impact physicians’ analytical thinking, critical thinking, and decision-making skills.	12 (3.0%)	80 (20.0%)	94 (23.5%)	128 (32.0%)	86 (21.5%)	3.49	5
9. Artificial intelligence lacks contextual knowledge and ability to read social clues.	6 (1.5%)	46 (11.5%)	110 (27.5%)	156 (39.0%)	82 (20.5%)	3.66	4
10. Physicians/nurses lack the time to learn how to use complex artificial intelligence-based medical devices.	10 (2.5%)	68 (17.0%)	114 (28.5%)	166 (41.5%)	42 (10.5%)	3.41	6

**Table 3:** Ethical challenges of integrating artificial intelligence into clinical practice.

	Strongly Disagree	Disagree	Neutral	Agree	Strongly Agree	Mean	Rank
1. Security and safety: Patient privacy and data security may be inadequately addressed in the integration of artificial intelligence systems in hospital practices.	4 (1.0%)	58 (14.5%)	96 (24.0%)	176 (44.0%)	66 (16.5%)	3.61	5
2. Health equity: Bias in artificial intelligence tools may result in unfair healthcare delivery.	16 (4.0%)	70 (17.5%)	146 (36.5%)	124 (31.0%)	44 (11.0%)	3.28	7
3. Informed consent: Ensuring appropriate informed consent becomes challenging when medical professionals are unable to effectively explain the functioning of artificial intelligence medical devices to patients.	6 (1.5%)	40 (10.0%)	120 (30.0%)	196 (49.0%)	38 (9.5%)	3.55	6
4. Accountability and responsibility: There is a concern about who is responsible if artificial intelligence makes medical errors without healthcare professionals’ input.	2 (0.5%)	26 (6.5%)	118 (29.5%)	142 (35.5%)	112 (28.0%)	3.84	2
5. Data ownership and control: Determining who owns the medical data used to train artificial intelligence systems and how they can be ethically and legally shared or sold.	4 (1.0%)	26 (6.5%)	138 (34.5%)	140 (35.0%)	92 (23.0%)	3.73	4
6. Cross-border issues: As artificial intelligence in healthcare often involves international collaborations and data sharing, ethical challenges arise concerning regulatory differences and standards used for patient care.	4 (1.0%)	20 (5.0%)	130 (32.5%)	158 (39.5%)	88 (22.0%)	3.77	3
7. Cultural sensitivity: Artificial intelligence algorithms developed in one culture may not be appropriate or effective when applied to diverse populations with different cultural norms.	2 (0.5%)	16 (4.0%)	104 (26.0%)	176 (44.0%)	102 (25.5%)	3.90	1

**Table 4:** Group statistics for the AI concerns difference by position.

	Education Level	N	Mean	Std. Deviation	Std. Error Mean
Physicians/nurses concerns on AI	Physician	295	3.5156	0.56990	0.03318
	Nurse	105	3.5571	0.57208	0.05583

**Table 5:** Independent samples *t*-test for AI concerns difference by position.

		Levene’s Test for Equality of Variances		*t*-Test for Equality of Means							
		F	Sig.	*t*	df	Significance		Mean Difference	Std. Error Difference	95% Confidence Interval of the Difference	
						One-Sided p	Two-Sided p			Lower	Upper
Physicians/nursing concerns on AI	Equal variances assumed	0.167	0.683	−0.641	398	0.261	0.522	−0.04155	0.06483	−0.16900	0.08590
	Equal variances not assumed			−0.640	182.396	0.262	0.523	−0.04155	0.06495	−0.16969	0.08659

**Table 6:** Independent samples effect sizes.

		Standardizer ^a^	Point Estimate	95% Confidence Interval	
				Lower	Upper
MEAN_V1	Cohen’s d	0.57047	−0.073	0.296	0.150
	Hedges’ correction	0.57155	−0.073	−0.295	0.150
	Glass’s delta	0.57208	−0.073	−0.295	0.150

^a^ The denominator used in estimating the effect sizes. Cohen’s d uses the pooled standard deviation. Hedges’ correction uses the pooled standard deviation, plus a correction factor. Glass’s delta uses the sample standard deviation of the control (i.e., the second) group.

**Table 7:** Group statistics for the AI ethical challenges difference by position.

	Education Level	N	Mean	Std. Deviation	Std. Error Mean
Ethical challenges of AI	Physician	295	3.6581	0.65499	0.03814
	Nurse	105	3.6871	0.65198	0.06363

**Table 8:** Independent samples *t*-test for the AI ethical challenges difference by position.

		Levene’s Test for Equality of Variances		*t*-Test for Equality of Means							
		F	Sig.	*t*	df	Significance		Mean Difference	Std. Error Difference	95% Confidence Interval of the Difference	
						One-Sided p	Two-Sided p			Lower	Upper
Ethical challenges of AI	Equal variances assumed	0.184	0.668	−0.390	398	0.349	0.697	−0.02896	0.07434	−0.17512	0.11719
	Equal variances not assumed			−0.390	183.752	0.348	0.697	−0.02896	0.07418	−0.17532	0.11739

**Table 9:** Independent samples effect sizes.

		Standardizer ^a^	Point Estimate	95% Confidence Interval	
				Lower	Upper
MEAN_V2	Cohen’s d	0.65421	−0.044	−0.267	0.179
	Hedges’ correction	0.65544	−0.044	−0.266	0.178
	Glass’s delta	0.65198	−0.044	−0.267	0.178

^a^ The denominator used in estimating the effect sizes. Cohen’s d uses the pooled standard deviation. Hedges’ correction uses the pooled standard deviation, plus a correction factor. Glass’s delta uses the sample standard deviation of the control (i.e., the second) group.

## Data Availability

Data are available upon request from the corresponding author due to the privacy of participants.

## Supplementary Materials

The following supporting information can be downloaded at: https://www.mdpi.com/article/10.3390/nursrep15090309/s1.

## Public Involvement Statement

No public involvement in any aspect of this research.

## Guidelines and Standards Statement

This manuscript was drafted against the STROBE (Strengthening the Reporting of Observational Studies in Epidemiology) for the cross-sectional research. A complete list of reporting guidelines can be accessed via the equator network: https://www.equator-network.org/, accessed on 16 March 2025.
