# Supplementary material for: Ethical and Practical Considerations of Physicians and Nurses on Integrating Artificial Intelligence in Clinical Practices in Saudi Arabia: A Cross-Sectional Study
Source: Nurs Rep. 2025 Aug 25;15(9):309. doi: 10.3390/nursrep15090309 (PMC12472769; doi:10.3390/nursrep15090309)
Supplement: Supplementary file 1 [file nursrep-15-00309-s001.zip › nursrep-3739488-supplementary.pdf]

Supplementary Materials

Ethical and Practical Perspectives of Physicians and Nurses on Integrating Artificial Intelligence in Clinical Practice

Informed Consent Form

Dear participant,

Researchers from different hospitals and universities are carrying out a research project with the purpose of assessing physicians' and nurses' ethical and practical perspectives on integrating artificial intelligence in clinical practice.

Your participation in this research study is voluntary. You may choose not to participate. If you decide to participate in this research survey, you may withdraw at any time.

We would like to confirm that all information provided here will be kept confidential. All data will be stored in a password-protected electronic format. To help protect your confidentiality, the surveys will not contain information that will personally identify you, and it will be used only for research purposes.

The procedure involves filling out an online survey that will take approximately 10 minutes. Your participation in completing this survey is highly appreciated.

ELECTRONIC CONSENT: Please select your choice below.

Clicking on the "agree" button below indicates that:

1. You have read the above information, AND
2. You voluntarily agree to participate

If you do not wish to participate in the research study, please decline participation by clicking on the "disagree" button.

- o Agree
- o Disagree

Part 1. Demographic information

Gender:

- Female
- Male

Position:

- Physician
- Nurse

Type of institution:

- Governmental hospitals
- Military clinic
- Private practice

Location:

- Urban area
- Rural area

Part 2. Physicians' experience with artificial intelligence

Frequency of artificial intelligence use in clinical practice:

- Daily
- Weekly
- Occasionally
- Never

Are you willing to use "artificial intelligence" tools in your clinical practice?

- Yes
- No
- I don't know
- Not able to

Are you aware of the potential benefits of using artificial intelligence?

- Yes
- No

Are you aware of the potential concerns of using artificial intelligence?

- Yes
- No

Do you know there is an area for use AI in your specialty?

- Yes
- No
- I don't know

Part 3. Physicians / Nurses concerns about the integration of artificial intelligence in clinical practice

Definition: In healthcare, artificial intelligence refers to the use of computer algorithms and software to analyze complex medical data. Artificial intelligence can assist in the clinical assessment, management and diagnosis of diseases, suggesting treatments, and predicting patient outcomes based on large datasets. It aims to improve efficiency, accuracy, and personalized care in medical settings.
From the following statements please select your level of agreement, with the scale from 1-5, where 1: strongly disagree, and 5: strongly agree.

**Statements (10 items)**	Strongly agree	Agree	Neutral	Disagree	Strongly disagree
Artificial intelligence might not understand complex medical conditions as accurately as physicians and nurses do.	**5**	**4**	**3**	**2**	**1**
Artificial intelligence could reduce the roles that physicians / nurses traditionally play.	**5**	**4**	**3**	**2**	**1**
Physicians / Nurses might feel more stressed because of the additional demands of using technology/ AI.	**5**	**4**	**3**	**2**	**1**
Artificial intelligence could potentially weaken the relationship between patients and their treating team.	**5**	**4**	**3**	**2**	**1**
Not all physicians / nurses have the adequate skills to use artificial intelligence effectively	**5**	**4**	**3**	**2**	**1**
There is a concern that artificial intelligence-based system could be manipulated from outside (third party, hackers...etc.)	**5**	**4**	**3**	**2**	**1**
Artificial intelligence will worsen problems in healthcare such as over utilization of laboratory testing, overdiagnosis, and overtreatment.	**5**	**4**	**3**	**2**	**1**
The use of artificial intelligence may negatively impact physicians / nurses analytical thinking, critical thinking and decision-making skills.	**5**	**4**	**3**	**2**	**1**
Artificial intelligence lacks contextual knowledge and ability to read social clues.	**5**	**4**	**3**	**2**	**1**
Physicians / Nurses lack the time to learn how to use complex artificial intelligence -based medical devices.	**5**	**4**	**3**	**2**	**1**

Part 4: Ethical challenges of integrating artificial intelligence in clinical practice

From the following statements please select your level of agreement, with the scale from 1-5, where 1: strongly disagree, and 5: strongly agree.

**Statements (7 items)**	**Strongly agree**	**Agree**	**Neutral**	**Disagree**	**Strongly disagree**
**Security and Safety:** Patient privacy and data security may be inadequately addressed in the integration of artificial intelligence systems in hospital practices.	**5**	**4**	**3**	**2**	**1**
**Patients Equity:** Bias in artificial intelligence tools may result in unfair healthcare delivery.	**5**	**4**	**3**	**2**	**1**
Informed Consent: Ensuring appropriate informed consent becomes challenging when medical professionals are unable to effectively explain the functioning of artificial intelligence medical devices to patients.	**5**	**4**	**3**	**2**	**1**
Accountability and Responsibility: There is a concern on who is responsible if artificial intelligence makes medical errors without healthcare professionals' input.	**5**	**4**	**3**	**2**	**1**
Data Ownership and Control: Determining who owns the medical data used to train artificial intelligence systems and how it can be ethically and legally shared or sold.	**5**	**4**	**3**	**2**	**1**
Cross-border Issues: As artificial intelligence in healthcare often involves international collaborations and data sharing, which arise ethical challenges concerning regulatory differences and standards used for patient care.	**5**	**4**	**3**	**2**	**1**
Cultural Sensitivity: Artificial intelligence algorithms developed in one cultural may not be appropriate or effective when applied to diverse populations with different cultural norms	**5**	**4**	**3**	**2**	**1**
